# Genome-Based Analysis of the Potential Bioactivity of the Terrestrial *Streptomyces vinaceusdrappus* Strain AC-40

**DOI:** 10.3390/biology12030345

**Published:** 2023-02-21

**Authors:** Abdelrahman M. Sedeek, Israa Salah, Hasnaa L. Kamel, Mohamed A. Soltan, Eman Nour, Abdulrahman Alshammari, Muhammad Shahid Riaz Rajoka, Tarek R. Elsayed

**Affiliations:** 1 Department of Microbiology and Immunology, Faculty of Pharmacy, Sinai University—Kantara Branch, Ismailia 41522, Egypt; 2 Faculty of Pharmacy, Modern University for Technology and Information (MTI), Cairo 11585, Egypt; 3 Faculty of Organic Agriculture, Heliopolis University for Sustainable Development, Cairo 11785, Egypt; 4 Department of Pharmacology and Toxicology, College of Pharmacy, King Saud University, Post Box 2455, Riyadh 11451, Saudi Arabia; 5 School of Dentistry, University of Maryland, Baltimore, MD 21201, USA; 6 Department of Agricultural Microbiology, Faculty of Agriculture, Cairo University, Giza 12613, Egypt

**Keywords:** actinobacteria, antimicrobial activity, plant growth promotion, whole genome sequencing, *Streptomyces rochei* group, genomics

## Abstract

**Simple Summary:**

The problem of antimicrobial resistance represents a real danger to mankind. There is a crucial necessity to search for new antibiotics. The current advances in sequencing techniques and bioinformatics represent a critical tool for finding out the hidden biosynthetic capabilities within bacterial genomes and finding out novel active secondary metabolites. In this work, we managed to recover the *Streptomyces vinaceusdrappus* strain AC-40, a rhizobacterium with potent and broad-spectrum antimicrobial activity. We made whole genome sequencing and genome mining for the genes and gene clusters related to beneficial traits such as the production of secondary metabolites and plant growth promotion.

**Abstract:**

*Streptomyces* are factories of antimicrobial secondary metabolites. We isolated a *Streptomyces* species associated with the *Pelargonium graveolens* rhizosphere. Its total metabolic extract exhibited potent antibacterial and antifungal properties against all the tested pathogenic microbes. Whole genome sequencing and genome analyses were performed to take a look at its main characteristics and to reconstruct the metabolic pathways that can be associated with biotechnologically useful traits. AntiSMASH was used to identify the secondary metabolite gene clusters. In addition, we searched for known genes associated with plant growth-promoting characteristics. Finally, a comparative and pan-genome analysis with three closely related genomes was conducted. It was identified as *Streptomyces vinaceusdrappus* strain AC-40. Genome mining indicated the presence of several secondary metabolite gene clusters. Some of them are identical or homologs to gene clusters of known metabolites with antimicrobial, antioxidant, and other bioactivities. It also showed the presence of several genes related to plant growth promotion traits. The comparative genome analysis indicated that at least five of these gene clusters are highly conserved through rochei group genomes. The genotypic and phenotypic characteristics of *S. vinaceusdrappus* strain AC-40 indicate that it is a promising source of beneficial secondary metabolites with pharmaceutical and biotechnological applications.

## 1. Introduction

The overuse and misuse of the currently available antibiotics have led to a fast increase in the resistance to these antibiotics with a heavy impact on human public health and national economies. As the problem of antimicrobial resistance becomes more serious, finding novel antibiotics has become more urgent than it has ever been [1,2].

Actinobacteria are reactors for production of the active secondary metabolites [3]. Several natural products have been derived from actinomycetes [3,4]. Over the last few years, actinomycetes took place in the fields of biotechnology, pharmacy, and agriculture [5].

*Streptomyces* represents the biggest genus in the phylum of Actinobacteria. They are high GC content, Gram-positive, and spore-forming bacteria. This genus is highly represented in the environment. From different soil and marine ecosystems [6,7] to extreme environments such as deserts and volcanic environments, different species of *Streptomyces* have been found [8,9]. With over 600 known species, the *Streptomyces* is a genus greatly known for the production of active compounds. Several bioactive molecules have been previously isolated from *Streptomyces* species. Antibacterial, antifungal, antitumor, anti-inflammatory, and immunosuppressive are all reported activities of secondary metabolites from the *Streptomyces* species [10]. Genome mining indicated that the genome of a single *Streptomyces* species can harbor from 25 up to 70 biosynthetic gene clusters (BGCs). Most of these BGCs are silent under standard laboratory conditions [5].

*Streptomyces* produces a chemically diverse collection of secondary metabolites with antimicrobial activity. Polyketides, non-ribosomal peptides, siderophores, terpenes, alkaloids, lanthipeptides, and others are among the most common classes of *Streptomyces* antimicrobial secondary metabolites [11]. The first antimicrobial compounds to be isolated from *Streptomyces* were actinomycin and streptothricin in the first half of the 20th century. Currently, *Streptomyces* species are a source of several known antibiotics, such as streptomycin, tetracycline chloramphenicol, clindamycin, lincomycin, erythromycin, kanamycin, and many others [12]. From 2015, more than 70 *Streptomyces* species with over 170 novel bioactive metabolites were isolated from terrestrial ecosystems. Many of these compounds had antimicrobial activity even against multidrug-resistant pathogens such as krisynomycin (B and C), picolinamycin, nybomycin D, puromycin B–E, ulleungmycin (A and B), streptoone A, and others [5].

*Streptomyces* is the most bacterial genus to be isolated as plant-associated actinobacteria [13]. It was reported to play an important role in the biological control of plant pathogens by producing antimicrobial molecules that fight against plant-infectious microbes. Some of their metabolites act as active plant growth promoters [14]. This makes the plant-associated *Streptomyces* a very promising source to search for novel antimicrobial metabolites. Some *Streptomyces* have also the capacity to produce enzymes with extracellular cell wall-degrading activity such as chitinases and β-1,3-glucanase that increases the plant’s resistance to diseases [15].

In this work, we aimed to investigate the genomic characteristics and the biosynthetic gene clusters of the biologically active *Streptomyces vinaceusdrappus* strain AC-40 that was isolated from the rhizosphere of the *Pelargonium graveolens* plant.

## 2. Materials and Methods

### 2.1. Isolation and Purification of the Strain AC-40

The strain AC-40 was isolated from the rhizosphere of three-month-old *Pelargonium graveolens*. A total of 5 g of *Pelargonium graveolens* root sample was placed in falcon tubes with 45 mL of 0.85% NaCl and vigorously mixed using a vortex for 1 min. Tenfold serial dilutions of the microbial suspensions were plated onto starch casein agar medium (SCA) supplemented with nystatin (100 µg/mL) to prevent fungal growth. The plates were incubated at 30 °C for 7 days. Typical actinomycetes colonies were picked and purified by streaking onto the SCA medium for 7 more days until full growth was observed. The pure colonies were preserved in Luria–Bertani (LB) broth medium supplemented with 20% glycerol at −20 °C for future use.

### 2.2. Solvent Extraction of the Strain AC-40 Bioactive Metabolites

Submerged fermentation with starch casein broth (SCB) medium was used to produce metabolites of the strain AC-40. A single colony of the isolated strain was inoculated in 250 mL of sterilized SCB medium and kept on a rotary shaker at 150 rpm, and 30 °C for 7 days. After fermentation, the supernatant was centrifuged at 2325× *g* (4000 rpm) for 15 min to remove the cell mass, and the crude cell-free supernatant was filter-sterilized using 0.22 µm filters. An equal volume of ethyl acetate was used to extract the cell-free supernatant. The top organic layer was collected in a beaker and evaporated at 30 °C using a rotor evaporator.

### 2.3. Antimicrobial Activity Screening of the Strain AC-40 Bioactive Metabolites

The antimicrobial activity of the cell-free supernatant of the broth culture and the ethyl acetate extract of the strain AC-40 was assessed against *Staphylococcus aureus* ATCC 6538, MRSA, *Bacillus cereus* ATCC 33018, *Pseudomonas aeruginosa* ATCC 27853, *Escherichia coli* (O157:H7), and *Candida albicans* ATCC 60193. Antimicrobial activity screening was analyzed using the agar well diffusion method on Mueller–Hinton agar (MHA) medium as follows: Bacterial and fungal pathogens (100 µL) were inoculated over the surface of MHA plates, 9 mm wells were prepared using a sterilized cork borer. The residual material resulting after the drying of the ethyl acetate extract of AC-40 was dissolved in 10% dimethyl sulfoxide (DMSO) with a concentration of 10 mg/mL then 50 µL was used in each well. An amount of 10% DMSO was used as a negative control, and gentamycin of a concentration of 10 μg/mL was used as a positive control for both Gram-positive and Gram-negative bacterial pathogens. Itraconazole of a concentration of 10 μg/mL was used as a positive control for fungal microbial strains. All assays were performed in triplicates.

### 2.4. Genomic DNA Extraction, and Sequencing of the Strain AC-40

A previously incubated SCB culture of the strain AC-40 at 30 °C for 7 days was used for genomic DNA extraction. A cell pellet of 1–5 × 10^6^ cells was prepared by centrifugation for 10 min at 5000× *g* (7500 rpm). The genomic DNA was extracted using the QIAamp^®^ DNA Minikit (QIAGEN, Hilden, Germany) following the manufacturer’s instructions. The purity of the extracted DNA was determined using NanoDrop 1000 Spectrophotometer V3.8 (Thermo Fisher Scientific Inc., Waltham, MA, USA). A260/A280 ratio of ~1.8 indicated a pure DNA extraction. The genome was sequenced using the Illumina MiSeq platform at the Genomics unit (57357, Egypt), following the standard Illumina protocols. The preparation of the library was carried out utilizing the Nextera XT DNA Library preparation kit (Illumina, San Diego, CA, USA).

### 2.5. Genome Assembly, Scaffolding, and Annotation of the Strain AC-40

The paired-ended reads with an average length of 277 bases were filtered using Trimmomatic, version 0.38.1 [16], with an adaptor cutting option, a sliding window of 4 bases, and a minimum quality of 20. The reads were assembled using the Unicycler version 0.4.8 assembler [17] on Pathosystems Resource Integration Center (PATRIC) server (www.patricbrc.org (accessed on 1 July 2022)), currently (https://www.bv-brc.org (accessed on 1 July 2022)). A minimum contig size of 1000 bp was used. For the reference-guided genome scaffolding of the draft genome, the similar genome finder (a part of PATRIC services) was used to compute genome distance estimation using Mash/MinHash algorithm [18] with all public genomes on the PATRIC platform. The most similar complete genome was used as a reference for the reference-guided genome scaffolding carried out using RagTag version 2.1.0 [19]. The genome was annotated using rapid annotations using subsystems technology (RAST) [20] and the rapid prokaryotic genome annotation (Prokka) [21].

### 2.6. Strain AC-40 Typing and Phylogeny

For typing the strain AC-40, the Type Strain Genome Server (TYGS) was used [22]. The phylogenetic tree was constructed using a concatenated sequence of the 16S rRNA gene sequence (~1500 pb), *gyrB* (~2000 pb), and *rpoB* (~1700 pb) sequences of the strain AC-40 and its most closely related type strains were aligned using MUSCLE [23]. The maximum likelihood phylogenetic tree was constructed using the Molecular Evolutionary Genetics Analysis software (MegaX) [24]. The Kimura 2-parameter model [25] and 1000-bootstrap test were used. The average nucleotide identity (ANI) of the genomes was calculated using the JSpecies Web Service [26]. In silico DNA–DNA hybridization (isDDH) of genomes was compared using the Genome-to-Genome Distance Calculator (GGDC) [27].

### 2.7. General Genome Feature and Pathways of the Strain AC-40

The annotated proteins from the genome of the strain AC-40 were analyzed on a subsystem level using the comprehensive genome analysis pipeline (a part of PATRIC serves). The GhostKOALA was used for KO annotation and reconstruction of the KEGG pathways [28].

### 2.8. Screening the Genome of the Strain AC-40 for Genes Related to Beneficial Traits

AntiSMASH bacterial version 6.0 [29] was used to scan the genome of *S. vinaceusdrappus* strain AC-40 for the presence of biosynthetic gene clusters (BGCs) responsible for the biosynthetic process of bioactive secondary metabolites. Furthermore, genes successfully annotated by Prokka were manually screened for gene products with plant growth-promoting (PGP) traits, such as genes incorporated in nitrogen fixation, phosphate solubilization, iron sequestration, and phytohormones production. We also searched for gene products that serve as biocatalysts, such as proteases, lipases, chitinases, and catalase production [30].

### 2.9. Pan-Genome and Comparative Genome Analysis

To identify the number of core genes, accessory genes, and the gene presence–absence distribution between the genome of the strain AC-40 and other genomes of the same phylogenetic group, a pan-genome analysis was conducted using Roary pan-genome pipeline [31].

The proteome comparison tool on PATRIC was used to compare the percentage identity between the protein sequences from other genomes referenced to those of the AC-40. The orthovenn2 web server was used to visualize and compare the orthologous protein clusters across these genomes [32].

The different BGCs detected with AntiSMASH in the genome of AC-40 were compared to the other genomes in the same phylogenetic group to investigate their conservation through the group.

## 3. Results

### 3.1. General Features and Antimicrobial Activity of the Strain AC-40

**Figure 1:**
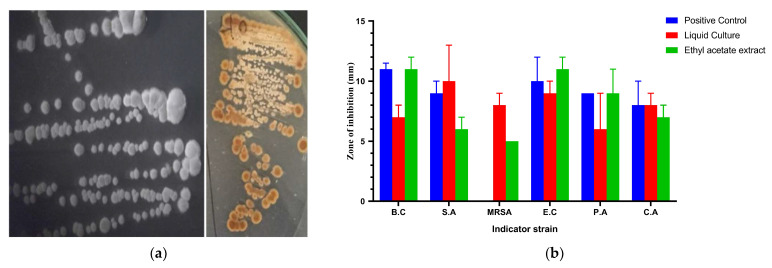
(**a**) The colony morphology of the strain AC-40 on starch casein agar; (**b**) zone of inhibition in (mm) produced by the cell-free supernatant and ethyl acetate extract of the strain AC-40 against B.C (*Bacillus cereus*), S.A (*Staphylococcus aureus*), MRSA (methicillin-resistant *Staphylococcus aureus*), E.C (*Escherichia coli*), P.A (*Pseudomonas aeruginosa*), and CA (*Candida albicans*). An amount of 10% DMSO was used as a negative control, and gentamycin (10 μg/mL) was used as a positive control.

The culture of the strain AC-40 on SCA, after 7 days, appeared as raised, rough, buff colonies. The culture had the distinctive earthy smell of geosmin-producing actinomycetes (Figure 1a). The catalase activity of the culture was positive. The cell-free supernatant and the ethyl acetate extract of strain AC-40 showed broad-spectrum antimicrobial activity against all indicator strains (Figure 1b). Both the cell-free liquid culture and total metabolic extract showed inhibitory effects significantly equivalent and sometimes higher than the positive control. This makes the AC-40 a potential source of antibacterial and antifungal compounds.

### 3.2. AC-40 Genome Assembly, Scaffolding, and Annotation

**Figure 2:**
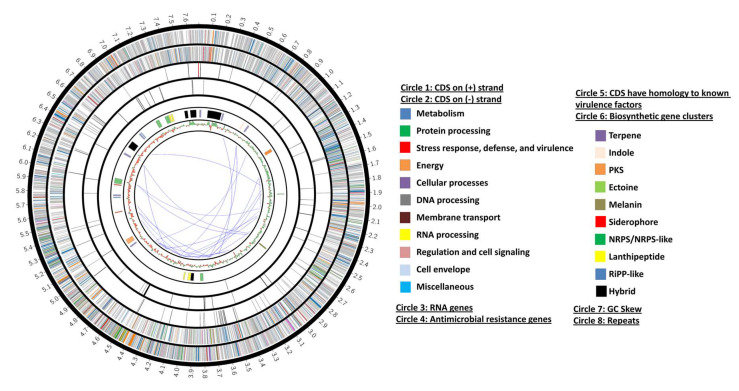
A circos diagram represents the genome map of the strain AC-40’s bacterial chromosome indicating the RAST, AntiSMASH annotations, GC Skew, and the repeated regions.

After the genomic DNA extraction, sequencing, and the de novo assembly of the genome of the strain AC-40, a total of 1048 contigs covering 7,493,232 bp were obtained. The average GC% was 72.41. Using the similar genome finder tool, the genome of *Streptomyces* sp. Osf17 (7,967,258 bp) was found to be the closest genome to the genome of strain AC-40 with a distance of 0.00944662. The genome of *Streptomyces* sp. Osf17 (GCA_019029505.1) is a two contigs genome (7,883,883 bp and 83,375 bp) sequenced with the PacBio Sequel technology. By using the genome of *Streptomyces* sp. Osf17 as a reference for the reference-guided scaffolding, one scaffold representing the bacterial chromosome was obtained. Genome annotation was conducted using two different platforms. The number of annotated CDSs was higher in the case of the RAST platform than it was in Prokka. A total of 7241 CDS (5059 functional), 35 transfer RNA (tRNA) genes, and 3 ribosomal RNA (rRNA) genes were annotated with RAST. A total of 6983 CDS (3303 functional), 82 tRNA genes, and 3 rRNA genes were annotated with Prokka. The genome map is illustrated in Figure 2.

### 3.3. AC-40 Typing and Phylogeny

The TYGS indicated a close relationship between the genome of AC-40 and *Streptomyces* species. The top hit was *Streptomyces vinaceusdrappus* (*Streptomyces rochei* group).

**Figure 3:**
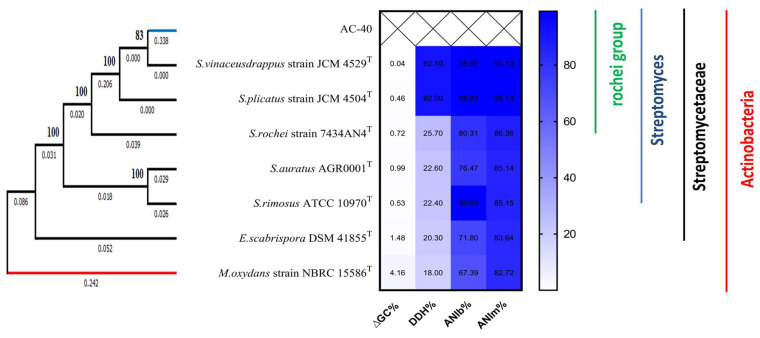
A 1000 bootstrap, un-rooted, maximum likelihood phylogenetic tree based on the 16S rRNA, *gyrB*, and *rpoB* gene partial sequences between *Streptomyces vinaceusdrappus* strain AC-40 and the closest related Streptomyces type strains; *S. vinaceusdrappus* strain JCM 4529, *S. plicatus* strain JCM 4504, *S. rochei* strain 7434AN4, *S. auratus* AGR0001, *S. rimosus* ATCC 10970, and *Embleya scabrispora* DSM 41855. *Microbacterium oxydans* strain NBRC 15586 was used to root the tree. The branch lengths are displayed under the branch. The bootstrap values are displayed beside the nodes.

The phylogenetic tree based on the 16S rRNA, *gyrB*, and *rpoB* gene partial sequences located the AC-40 within the *S. rochei* group (Figure 3). The ANI, DDH, and ∆GC% values comparisons between other genomes and the genome of AC-40 were calculated to reinforce the strain typing. We identified the isolate AC-40 as *S. vinaceusdrappus* strain AC-40.

### 3.4. Streptomyces vinaceusdrappus Strain AC-40 Genome Features and Pathways Reconstruction

**Figure 4:**
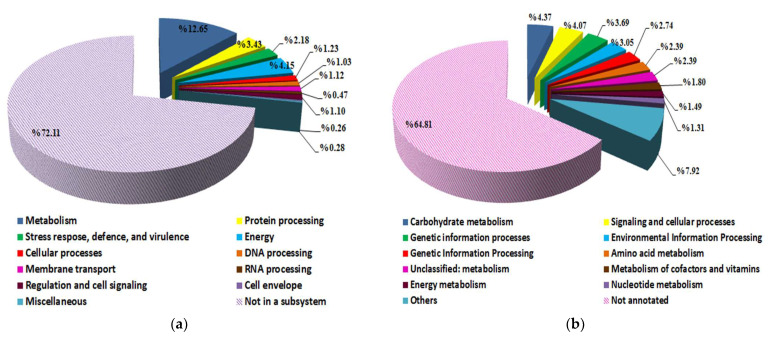
(**a**) a subsystem analysis by PATRIC platform indicates the number of genes incorporated in specific biological process within the genome of *S. vinaceusdrappus* strain AC-40; (**b**) a functional categorization of KEGG annotations of the genome of *S. vinaceusdrappus* strain AC-40 using the GhostKOALA tool.

A subsystem is a group of proteins that work together to carry out a certain biological function or structural complex. PATRIC comprehensive genome analysis service provides a subsystem analysis of prokaryotic genomes. The subsystem analysis of *S. vinaceusdrappus* strain AC-40 showed that from the 7241 annotated CDS only 2023 CDS were assigned as “in a subsystem” (Figure 4a). A total of 2530 protein products distributed over 11 functional categories were annotated on KEGG using the GhostKOALA tool (Figure 4b). The 2530 annotated gene products are involved in 327 pathways over 59 complete KEGG modules, including carbohydrate metabolism (13 modules), carbon fixation (2 modules), nitrogen metabolism (1 module), sulfur metabolism (1 module), ATP synthesis (5 modules), lipid metabolism (3 modules), nucleotide metabolism (9 modules), amino acid metabolism (13 modules), cofactors and vitamins biosynthesis (10 modules), terpenoids and polyketides (2 modules). Among the metabolic modules that were found complete within the genome of the *S. vinaceusdrappus* strain AC-40 were the dissimilatory nitrate reduction to ammonia, the assimilatory sulfate reduction, and crassulacean acid metabolism.

### 3.5. Streptomyces vinaceusdrappus Strain AC-40 Secondary Metabolites-Related Gene Clusters

A total of 27 biosynthetic gene clusters (BGCs) were detected within the genome of *S. vinaceusdrappus* strain AC-40 using AntiSMASH bacterial version 6.0 (Table 1).

**Figure 5:**
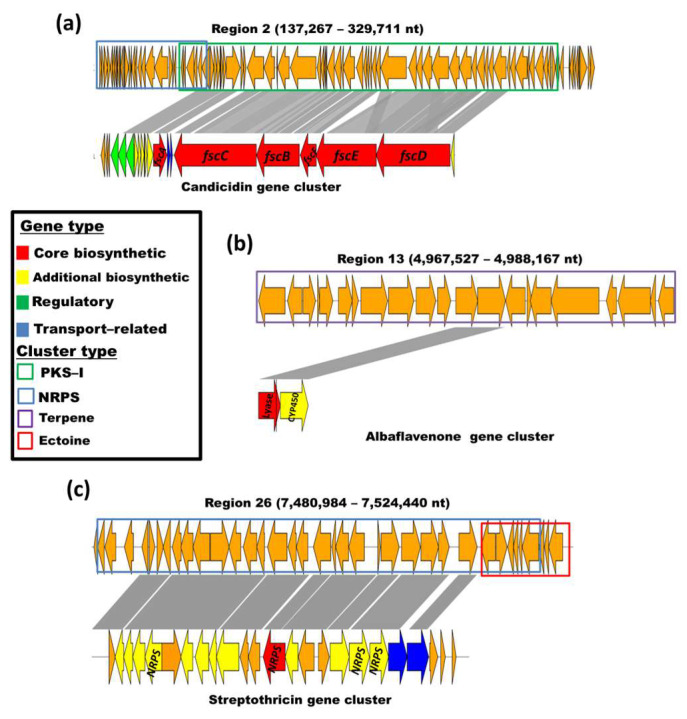
Similarity to known antimicrobial metabolites BGCs in three of the regions detected with AntiSMASH in the genome of *Streptomyces vinaceusdrappus* strain AC-40: (**a**) candicidin; (**b**) albaflavenone; (**c**) streptothricin. The characteristics of the clusters and gene types are indicated in the box.

Some of the BGCs were highly related to known metabolites with antibacterial and antifungal properties such as candicidin, albaflavenone, and streptothricin (Figure 5). Other clusters related to known active metabolites such as the carotene derivative isorenieratene, ectoine, hopene, coelichelin, coelibactin, desferrioxamine, SapB, and geosmin were detected. Some of the clusters showed small or no relevance to known metabolites. The type of these clusters included terpene, T2PKS, NRPS, RiPP-like, lanthipeptides, siderophores, or hybrid gene clusters.

### 3.6. Streptomyces vinaceusdrappus Strain AC-40 Genes Related to Plant Growth Promotion Traits

The functional annotation by Prokka revealed the presence of several genes within the genome of *S. vinaceusdrappus* strain AC-40 whose annotated products can have PGP characteristics (Table 2). Those genes are related to processes that play a helpful role in plant nutrition, such as nitrogen assimilation, phosphate solubilization, and iron sequestration via siderophores. Chitin is the major component of the cell wall of several phytopathogens, especially fungi [33]. A set of genes responsible for the production of chitinases and other lytic enzymes such as proteases and lipases were detected within the genome of the *S. vinaceusdrappus* strain AC-40. That suggests a strong antifungal activity of this strain. The production of catalases and peroxidases by rhizobacteria is a very helpful mechanism in plant oxidative stress tolerance [33]. A set of genes responsible for the peroxidase/catalase activity were also detected within the genome of the AC-40.

### 3.7. Pan-Genome and Comparative Genomics

**Figure 6:**
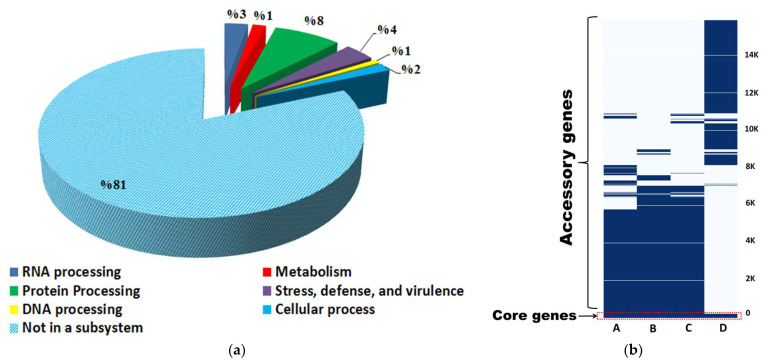
(**a**) Subsystem analysis of the rochei group’s core genome indicates the number of genes incorporated in specific biological process; (**b**) gene presence–absence matrix in the four genomes A (*S. vinaceusdrappus* strain AC-40), B (*S. vinaceusdrappus* strain JCM 4529), C (*S. plicatus* strain JCM 4504), and D (*S. rochei* strain 7434AN4).

The pan-genome analysis of the *S. vinaceusdrappus* strain AC-40 and other related three members of *S. rochei* group genomes (*S. plicatus* strain JCM 4504 (GCA_014650135.1), *S. vinaceusdrappus* strain JCM 4529 (ASM1465021v1), and *S. rochei* strain 7434AN4 (GCA_008064995.1) showed the presence of 246 core genes shared within the four genomes and 15638 accessory genes distributed within them. Most of the core genes are incorporated in protein processing, cell defense, and stress response (Figure 6a). The KEGG mapper reconstruction of the core indicated that these genes are incorporated in 90 different pathways but there was only one complete central carbohydrate metabolism module representing the phosphoribosyl-diphosphate (PRPP) biosynthesis in prokaryotes. The gene presence–absence matrix showed a unique print of the AC-40 genome which was more related to genomes of *S. plicatus* strain JCM 4504 and *S. vinaceusdrappus* strain JCM 4529 than the genome of *S. rochei* strain 7434AN4 (Figure 6b).

**Figure 7:**
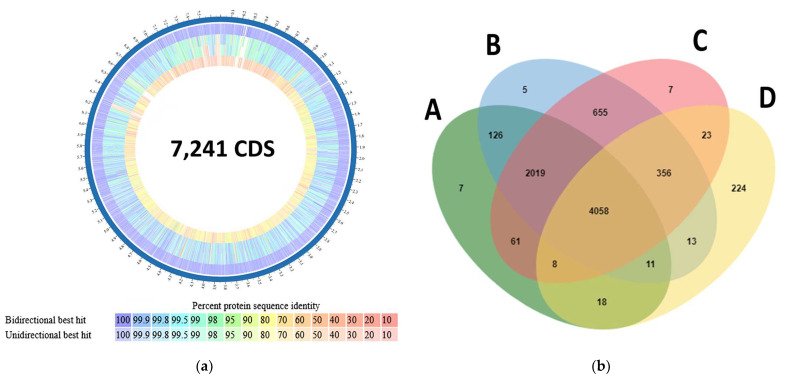
(**a**) Proteome similarity analysis carried out by the proteome comparison tool on PATRIC between—as ordered from the outside to the inside—*S. vinaceusdrappus* strain AC-40 as a reference, *S. vinaceusdrappus* strain JCM 4529, *S. plicatus* strain JCM 4504, and *S. rochei* strain 7434AN4; (**b**) A Venn diagram indicated the shared orthologous protein clusters among A (*S. vinaceusdrappus* strain AC-40), B (*S. vinaceusdrappus* strain JCM 4529), C (*S. plicatus* strain JCM 4504), and D (*S. rochei* strain 7434AN4).

The proteome comparison tool on PATRIC showed that most of the proteins in *S. vinaceusdrappus* strain JCM 4529, and *S. plicatus* strain JCM 4504 had similarity >90% to those of *S. vinaceusdrappus* strain AC-40. However, S. *rochei* strain 7434AN4 showed similarities of 80% or lower on the protein level (Figure 7a). The orthologous cluster protein analysis showed that 4058 clusters are shared between all four genomes. Only seven protein clusters (26 protein sequences) were unique to the genome of AC-40 and not annotated on Swiss-Prot (Figure 7b).

Comparing the biosynthetic genes clusters between the four genomes, most of the clusters were found to be conserved among *S. vinaceusdrappus* strain AC-40, *S. vinaceusdrappus* strain JCM 4529, and *S. plicatus* strain JCM 4504 with few differences. Region 11, a Lanthipeptide class-V BGC, was found within the genome of the *S. vinaceusdrappus* strain AC-40. This cluster showed no relevance to any known clusters. Instead, within the genomes of *S. vinaceusdrappus* strains JCM4529 and *S. plicatus* strain JCM4504, there was a hybrid gene cluster of the type PKS-like/furan/lanthipeptide class-v. The BGC in region 26 within the genome of the *S. vinaceusdrappus* strain AC-40 was not detected within the genome of *S. plicatus* strain JCM4504. This gene cluster was highly similar to that of the known antimicrobial metabolite streptothricin. Region 27, representing the hybrid PKS-II/butyrolactone cluster within the genome of *S. vinaceusdrappus* strain AC-40, was not found within the genomes of *S. vinaceusdrappus* strains JCM4529 and *S. plicatus* strain JCM4504. However, the sequence of the PKS-II part of the cluster was highly similar to a single PKS-II gene cluster found within the genomes of *S. vinaceusdrappus* strains JCM4529 and *S. plicatus* strain JCM4504.

**Figure 8:**
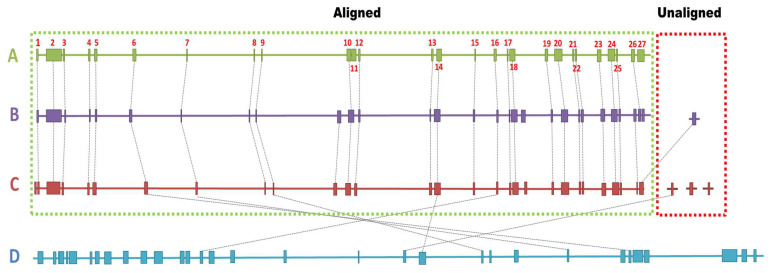
A relation between 27 biosynthetic gene clusters among A (*S. vinaceusdrappus* strain AC-40) as a reference, B (*S. vinaceusdrappus* strain JCM 4529), C (*S. plicatus* strain JCM 4504), and D (*S. rochei* strain 7434AN4). The labels aligned and unaligned indicate the contigs from genomes B and C that were successfully and unsuccessfully aligned on the genome A as reference for reference-guided scaffolding.

*S. rochei* strain 7434AN4 had the lowest relation to the BGCs in the *S. vinaceusdrappus* strain AC-40 (Figure 8).

## 4. Discussion

Rhizobacteria play an important role in plant growth under both normal and challenging environmental circumstances [30]. Those bacteria also play an important role in the biocontrol process against plant pathogens by producing antimicrobial metabolites [34]. The interaction between the plant and its associated bacteria can also take a deeper connection where some endophytic bacteria can synthesize one or more of their host’s metabolites [35].

AC-40 was isolated from the rhizosphere of *Pelargonium graveolens,* a medicinal, aromatic plant belonging to the family Geraniaceae. It is known for its antibacterial, antifungal, and antioxidant activities [36].

The closely related genome of *Streptomyces* sp. Osf17 which showed the lowest genomic distance between all publicly available genomes on PATRIC has been isolated from the Algerian desert and was reported for its potent antimicrobial activity [9]. This may suggest the ability of AC-40 to survive in extreme conditions. The genome of AC-40 showed the presence of several genes responsible for stress response, including those of the *sigmaB* stress response regulator.

Phylogenetic analysis showed that the isolate is closely grouped with *S. vinaceusdrappus* (*S. rochei* group). Most of the members of the rochei group were reported as antibiotic-producing *Streptomyces* [37,38]. The genomes of this group of *Streptomyces* are understudied. The genome list on NCBI of the members from this group is quite short. The *S. vinaceusdrappus* and *S. plicatus* genome list contain only one genome for each.

The reconstruction of the KEGG pathways indicated the presence of some interesting, complete modules. The dissimilatory nitrate reduction to ammonia is a beneficial ability for the microorganism and plant. It converts the nitrate and nitrite compounds into the less mobile ammonia compound. It serves for longer availability of nitrogen for both the microorganism and plant [39]. The hydrogen sulfide produced from the act as a signaling molecule that can promote plant growth and germination [40]. Crassulacean acid metabolism (CAM) is believed to be another mode of photosynthesis in some plants that have been found long ago as an evolutionary response to challenging climate conditions [41]. Half of this mode takes place in the dark and the other half during the day. Interestingly, we have found a complete set of genes code for the CAM pathway’s enzymes in the dark and a nearly complete set of its enzymes during the day in our *Streptomyces* sp. AC-40. We have also found this to be common in several public genomes belonging to different species from the *Streptomyces* genus. Liu et al. (2016) have described the same notice in the sponge-active symbiotic filamentous bacteria of the genus “*Entotheonella*” (a phylum of Tectomicrobia) [42].

The AntiSMASH results indicated the presence of several BGCs. The largest number of those BGCs was of the type of NRPS and terpenes. PKS-I and PKS-II are significantly represented in the terrestrial and aquatic environments and strongly related to the biosynthesis of antimicrobial metabolites in actinobacteria, especially the *Streptomyces* species [43]. Six of the PKS and PKS-like gene clusters were found within the genome of AC-40.

At least three of the detected gene clusters are related to known potent antimicrobials. Streptothricin is an N-glycoside antibiotic that has been found in different *Streptomyces* species with broad-spectrum activity against both Gram-positive and Gram-negative bacteria [44]. Candicidin is a polyene antifungal originally derived from *Streptomyces griseus* and clinically approved against *Candida albicans* vaginal infections [45]. Albaflavenone is a sesquiterpene antibiotic originally derived from *Streptomyces coelicolor* A3(2) [46]. These results can explain the potent and broad antimicrobial activity of *S. vinaceusdrappus* strain AC-40. The same results were reported from the closely related *Streptomyces* sp. Osf17 [9].

Other BGCs are related to other active metabolites, including isorenieratene, which was reported for its strong antioxidant and protection against photooxidation ultraviolet wave damage [47]. Ectoine is a bacterial natural osmolyte produced by many extremophiles. It plays an important role in bacterial survival in high osmotic environments. Ectoine was reported for its ability to protect the biological membrane (e.g., skin) from extreme conditions such as dryness, heat, UV, and surfactants [48,49]. With three siderophore BGCs, the siderophore peptide “coelichelin” and the zincophore “coelibactin”, which are detected in our strain and other related strains, are thought to be highly adapted to limited-nutrient conditions [50,51].

Lanthipeptides are RiPPs that produce a wide range of bioactivities [52]. Class I and II lanthipeptides were usually reported for their broad antimicrobial activity through binding to lipid II, inhibiting cell wall synthesis [53]. There was a complete lanthipeptide class I lanthipeptide gene cluster detected within our isolate AC-40 genome with no relevance to any known lanthipeptide, suggesting its novelty. Unlike classes I and II lanthipeptides, classes III and V usually play biological functions [53]. *S. vinaceusdrappus* strain AC-40 showed the presence of two lanthipeptides class III gene clusters. One of them belongs to the known lanthipeptide *SapB*. It plays an important morphogenetic function in the formation of aerial mycelium [53,54].

## 5. Conclusions

Rhizoactinobacteria are good producers of antimicrobial secondary metabolites for self-defense and competing with other microbes for space and nutrients, including the host-pathogenic ones. This represents a bright side for the microorganism–host interaction.

The rhizobacterium *S. vinaceusdrappus* strain AC-40 is an active producer of antimicrobial secondary metabolites. Its genome is quite interesting for carrying several secondary metabolite-related gene clusters which have little or no relation to known ones, which make it a potential producer for novel antimicrobial metabolites. Some of those gene clusters were found to be conserved or semi-conserved in the *S. rochei* group, which make this group interesting for deeper investigation.

## Figures and Tables

**Table 1:** The biosynthetic gene clusters (BGCs) detected with AntiSMASH within the genome of *Streptomyces vinaceusdrappus* strain AC-40.

Region	Start	End	BGC Type	Most Similar Known Metabolite	%Identity
1	11,426	34,005	Terpene	Isorenieratene	85%
2	137,267	329,711	PKS-I/NRPS	Candicidin	95%
3	347,918	368,491	Terpene	Lysolipin I	4%
4	669,221	690,342	Indole	5-dimethylallylindole-3-acetonitrile	100%
5	748,998	771,393	Terpene	Carotenoid	54%
6	1,220,910	1,262,076	PKS-III	Flaviolin/1,3,6,8-tetrahydroxynaphthalene	100%
7	1,898,364	1,907,075	Ectoine	Ectoine	100%
8	2,737,184	2,747,792	Melanin	Istamycin	4%
9	2,831,522	2,842,444	Siderophore	Desferrioxamin B and E	83%
10	3,919,974	3,960,930	PKS-like/furan	Methylenomycin A	9%
11	3,962,785	4,005,126	Lanthipeptide-V	Cacaoidin	7%
12	4,052,201	4,071,203	Lanthipeptide-III	Catenulipeptin	60%
13	4,967,527	4,988,167	Terpene	Albaflavenone	100%
14	5,025,751	5,094,254	PKS-II	Spore pigment	66%
15	5,510,542	5,520,672	Siderophore	-	-
16	5,755,125	5,776,362	Terpene	Geosmin	100%
17	5,916,847	5,928,346	Siderophore	Grincamycin	8%
18	5,942,866	6,016,271	NRPS	Lipopeptide	72%
19	6,380,102	6,406,068	Terpene	Hopene	100%
20	6,500,253	6,601,623	PKS-I	Vicenistatin	80%
21	6,739,417	6,760,448	Terpene	Versipelostatin	5%
22	6,773,508	6,783,723	Ripp-like	Informatipeptin	42%
23	7,035,002	7,091,898	NRPS	Coelichelin	100%
24	7,186,423	7,269,342	NRPS/Lanthipeptide-I	Coelibactin	100%
25	7,280,111	7,302,690	Lanthipeptide-III	SapB	100%
26	7,480,984	7,524,440	NRPS/Ectoine	Streptothricin	87%
27	7,563,953	7,649,675	PKS-II/Butyrolactone	Fluostatins M-Q	60%

**Table 2:** Some genes related to the plant growth promotion and their representation within *Streptomyces vinaceusdrappus* strain AC-40 according to Prokka annotations.

Trait	Genes	Product
Nitrogen assimilation	*nasD*, *nirD*	Nitrite reductase
	*nark*	Nitrate/nitrite transporter
Phosphate solubilization	*gpm2*	Acid phosphatase
	*phoD*	Alkaline phosphatase D
	*ycdX*	Phosphatase
	*-*	Putative phosphatase
Iron sequestration	*yfiZ*, *yfhA*	putative siderophore transport system permease protein
	*yusV*	putative siderophore transport system ATP-binding protein
Phytohormone synthesis	*trpC*	Indole-3-glycerol phosphate synthase
Biocatalyst	*htpX*, *prtS*	Protease
	*snpA*	Extracellular small neutral protease
	*ydeA*	Putative protease
	*lip1*	Lipase 1
	*-*	Lipase 2
	*-*	Thermostable monoacylglycerol lipase
	*chiA*	putative bifunctional chitinase/lysozyme
	*chiC*, *chiD*, *chtA*	Chitinase
	*-*	Exochitinase 1
	*aml*	Alpha-amylase
	*katA*, *katE*	Catalase
	*bca*	Bromoperoxidase-catalase
	*katG*	Catalase-peroxidase

## Data Availability

The data were uploaded to the NCBI GenBank under the accession numbers; BioProject (PRJNA879080), BioSample (SAMN30790593), SRA (SRR21521741), and Genome (CP104697).
